# Caring for people living with, and beyond, cancer: an online survey of GPs in England

**DOI:** 10.3399/bjgp15X687409

**Published:** 2015-10-26

**Authors:** Fiona M Walter, Juliet A Usher-Smith, Suresh Yadlapalli, Eila Watson

**Affiliations:** Department of Public Health and Primary Care, University of Cambridge, Cambridge.; Department of Public Health and Primary Care, University of Cambridge, Cambridge.; Department of Public Health and Primary Care, University of Cambridge, Cambridge.; Department of Clinical Health Care, Oxford Brookes University, Oxford.

**Keywords:** cancer, follow up-care, general practitioners, primary health care, survivors, views

## Abstract

**Background:**

Increasing numbers of people are living with, and beyond, cancer. They are at risk of long-term morbidity and premature mortality due to the consequences of their disease and its treatment. Primary care can contribute to providing ongoing care.

**Aim:**

To determine the current practice and views of GPs in England regarding cancer survivorship care.

**Design and setting:**

Online survey of a sample of 500 GPs, stratified by NHS region in England.

**Method:**

The survey included questions adapted from prior surveys assessing physician knowledge and attitudes regarding care of patients with cancer.

**Results:**

In total, 500 GPs responded; approximately half reported often providing care to people living beyond cancer for treatment-related side effects (51%), psychological symptoms (65%), and lifestyle advice (55%). Only 29% felt very confident managing treatment-related side effects compared with 46% and 65% for psychological symptoms and lifestyle advice respectively. Half reported usually receiving cancer treatment summaries and survivorship care plans but most of the sample felt these would improve their ability to provide care (76%). Only 53% were convinced of the usefulness of cancer care reviews. Although most felt that primary and specialist care should share responsibility for managing bone (81%) and cardiovascular (77%) health consequences, fewer than half reported often taking previous history of cancer or cancer treatment into consideration when assessing bone health; only one-fifth did this in relation to cardiovascular health. Most responders were interested in receiving education to improve their knowledge and expertise.

**Conclusion:**

GPs have a potentially important role to play in caring for people following cancer treatment. This study has highlighted areas where further support and education are needed to enable GPs to optimise their role in cancer survivorship care.

## INTRODUCTION

As cancer treatments improve and people live longer, the number of individuals living with, and beyond, cancer is increasing. More than 80% of children and adolescents, and 50% of adults diagnosed with cancer, will survive for ≥5 years after completing their cancer treatment.^1^,^2^ There are currently >14.5 million US and 2 million UK survivors of cancer, equating to about 3% of each population; over the next decade these numbers are expected to grow to 19 million and 3 million respectively.^3^,^4^

Traditionally, follow-up care has been provided by cancer specialists and has mainly focused on monitoring for recurrence and second primary cancers. With the number of survivors of cancer growing dramatically, a model of care led purely by secondary care is clearly no longer feasible;^5^ a more holistic approach is also needed. Integrated models of care are warranted, as when care is shared between secondary and primary care, or in models in which primary care takes over the care of patients who are stable earlier than currently happens. The National Cancer Survivorship Initiative (NCSI) has proposed a risk-stratified approach, whereby those at low risk are supported to self-manage in the community, those at high risk are managed by specialist teams, and care for those at moderate risk is shared by secondary and primary care.^6^

Many survivors of cancer are at risk of chronic morbidity and premature mortality as a consequence of their cancer and its treatment.^7^ Some of these consequences present during, or soon after, active treatment and can persist for many years. They include:

- chronic fatigue;
- persistent pain;
- mental health problems;
- sexual dysfunction; and
- urinary and gastrointestinal problems.^8^

Others are considered to be ‘late effects’, due to the cancer and its treatment. These may include bone, pulmonary, renal, or neurological toxicities, and there is also increasing evidence that cardiovascular health can be affected by many types of cancer treatment. As an example, following childhood cancer treatment, people are five times more likely to develop congestive heart failure and myocardial infarction compared with their siblings.^9^,^10^

As many as 500 000 UK survivors of cancer are considered to be experiencing consequences attributable to cancer treatment,^4^ yet there is evidence that many, particularly adult survivors of childhood cancer, do not receive appropriate generalist or specialist care that focuses on the risks arising from their prior cancer therapy.^11^

**How this fits in**

As many as 500 000 survivors of cancer in the UK are considered to be experiencing consequences attributable to cancer and its treatment. Despite recent recommendations, only half the surveyed GPs in England reported usually receiving cancer treatment summaries and survivorship care plans, and fewer took a previous history of cancer or cancer treatment into consideration when assessing bone or cardiovascular health. The findings suggest that interventions should focus on improving communication between specialist and primary care, raising GP awareness of physical and psychological consequences, and enhancing GP knowledge of late effects of cancer treatment.

Over the last decade an international focus on cancer follow-up has begun to recognise these unmet needs of survivors of cancer.^5^,^12^ Experts have advocated the value of sharing survivorship care between specialists and primary care, and there is increasing evidence that, as well as preventive care, such as advice around physical activity and healthy weight management,^13^ follow-up care for some cancers can be provided as safely and effectively in primary as in secondary care.^14^,^15^ In the UK, primary care is potentially well placed to undertake this work, with its universal system of patient registration, generalist skills, and high satisfaction ratings.

The 2012 UK NCSI promoted a ‘recovery package’ that included a cancer treatment summary for patients and their GPs. It detailed possible treatment toxicities, late effects, and alert symptoms requiring referral back to specialist care, along with an ongoing management plan.^16^,^17^ It also recommended a survivorship care plan, a structured holistic needs assessment aiming to help assessment and monitoring, and a cancer care review to be undertaken by a GP within 6 months of their patient’s cancer diagnosis. This should be encouraged through inclusion in the general medical services Quality and Outcomes Framework.^18^

Against this background of recent recommendations, the aim of this study was to examine the current practices and views of GPs in England on providing care for those living with, and beyond, cancer. The researchers were interested in GPs’ experiences, knowledge, and views in relation not only to caring for people who have recently completed active cancer treatment, but also to caring for people living beyond cancer treatment in general, and in relation to cardiovascular and bone health after cancer specifically.

## METHOD

### Participants

A questionnaire was distributed electronically via Medix, a leading market research consultancy specialising in high-quality online research using pre-recruited panels of medical professionals. At the time of recruitment, the UK GP panel size was approximately 10 000. Sampling was restricted to currently practising GPs, was stratified by NHS region, and was conducted on a ‘first come, first served’ basis; the target number of responses was 500.

Recruitment started in mid-June 2014 and continued for 4 weeks, when the target number of responses was reached. Potential participants were sent an e-mail explaining the purpose of the study and a web link to the survey; entry into a prize draw for taking part was also offered.

### Survey instrument

The survey (available from the authors on request) was adapted from prior surveys assessing physician knowledge and attitudes regarding care of patients with cancer,^19^,^20^ and also included new questions generated specifically for this study and informed by expert opinion. It comprised 25 questions focused on follow-up care for survivors of cancer, who were defined as ‘people living with, and beyond, cancer (excluding non-melanoma skin cancer) or those who have completed definitive primary cancer treatment’.^21^ Quantitative survey items utilised ‘yes/no’ responses and five-point Likert scales. The questionnaire was piloted with non-academic GP colleagues (*n* = 6) to ensure face validity. No questions were removed or added in this process but the wording of some items was slightly modified in response to their feedback. For example, ‘further training’ was changed to ‘further learning opportunity’ and the word ‘diminished’ when referring to ‘diminished cardiovascular or bone health’ was amended to ‘reduced’.

The survey sought information about GP demographics, experience, workload, and practice type Responders were asked:

- about their management of people who had recently completed active cancer treatment, including how often they received cancer treatment summaries and survivorship care plans from hospital specialists, and what information was included;
- how often they provided specific care for these people, including management of treatment-related side effects, psychological symptoms, lifestyle factors, and advice concerning work and finances, and how confident they felt in this role;
- how frequently they conducted cancer care reviews, whether they found them useful, and how they were undertaken, including the use of templates;
- how often they were aware that a patient had been diagnosed with cancer within 5 years, 5–10 years ago, and >10 years ago;
- about their awareness of possible late cardiovascular and bone effects following treatment, and their opinion on who should be responsible for the management of these possible treatment consequences; and
- about training they had undertaken and would like to receive in this area.

The questionnaire included one open-ended question that allowed responders to provide comments about the training they had undertaken.

### Statistical analysis

Participants entered their survey responses online. Descriptive statistics were used to report practitioner characteristics, frequencies of current practice, and knowledge and training items. When comparing how often they considered a diagnosis of cancer, the responses were dichotomised into ‘never’, ‘rarely’, and ‘sometimes’ versus ‘often’. For their knowledge of associations between cancer treatment and reduced health, the proportion correctly answering each question was compared with those who answered incorrectly or did not know.

Reported modes of previous cancer education were grouped into categories:

- self-directed learning (reading, e-learning, consultant letters, or hospice newsletters);
- attending meetings or lectures;
- more formal courses (GP Update, or diploma course); and
- clinical attachments.

McNemar’s test was used to compare how often GPs considered a diagnosis of cancer when assessing cardiovascular or bone health, and logistic regression when comparing which features are associated with finding cancer care reviews useful.

All analyses were performed using Stata (version 12). Results are presented as odds ratios (ORs) and 95% confidence intervals (CIs). Statistical significance was set at *P* = 0.05.

## RESULTS

### Participant characteristics

Participant characteristics are shown in Table 1. There were 500 GP responders, who were drawn fairly equally from across the 10 NHS regions (East of England, London, South Central, North East, North West, Yorkshire and the Humber, East Midlands, West Midlands, South East Coast, and South West). GPs ranged from 37–59 (7–11%) per region. There were more male (75%) than female responders; most were GP partners in primary care practice (79%) with >15 years’ experience (73%). Just over half were working full time (54%) and almost one-quarter were GP trainers (23%).

**Table 1.** Characteristics of participants (*n* = 500)

**Characteristic**	***n* (%)**
**Sex**	
Male	377 (75)
Female	123 (25)

**Experience, years**	
<5	5 (1)
5–10	34 (7)
10–15	96 (19)
>15	365 (73)

**Type of GP**	
Partner	397 (79)
Salaried GP	75 (15)
Locum	25 (5)
Trainee	2 (0.4)
Trainer	117 (23)
Macmillan GP	9 (2)

**GP practice**	
Urban	269 (54)
Rural	78 (16)
Mixed	153 (31)

**GP workload**	
Half time (≤4 sessions/week)	63 (13)
Three-quarter time (5–7 sessions/week)	168 (34)
Full-time (≥8 sessions/week)	269 (54)

In this study, the participants comprised more male GPs, GP partners, and trainers, and fewer full-time workers, than the national average: 49% of GPs in England are male, 66% are GP partners, 16% are trainers, and 71% work full-time.^22^,^23^

### Recent active cancer treatment

**Figure 1.**
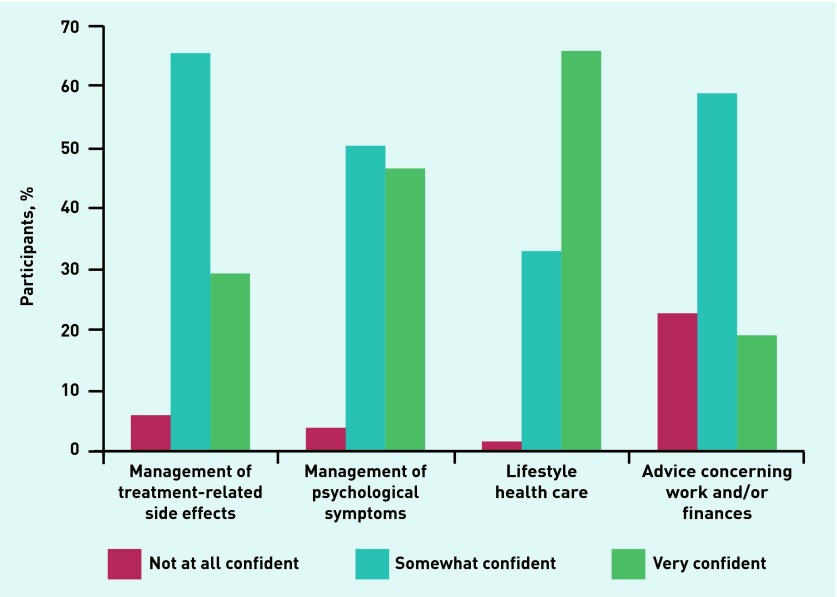
***GP confidence in provision of specific care to patients who recently completed cancer treatment, by care type.***

Approximately half of the GP responders reported often/almost always receiving a detailed cancer treatment summary, details of ongoing care from the hospital, or details of ongoing care to be provided by the GP. Only one-fifth often received information on when to refer back specialist care, and just under one-sixth received information on late effects (Table 2). Half the GPs reported often providing care relating to treatment-related side effects (51%), psychological symptoms (56%), and lifestyle (55%), but fewer often gave advice concerning work and/or finances (30%). This was reflected in their reported confidence in providing these aspects of care: GPs were most confident providing lifestyle advice or managing psychological symptoms and least confident managing work or financial issues (*P*<0.0005) (Figure 1).

**Table 2.** Care for people who recently completed active cancer treatment

	**Rarely/never/don’t know,^a^*n* (%)**	**Sometimes, *n* (%)**	**Often/almost always,^a^*n* (%)**
**How often the GPs receive:**			
A short treatment summary^a^	38 (7.6)	114 (22.8)	348 (69.6)
A detailed treatment summary^a^	89 (17.8)	160 (32.0)	251 (50.2)
Details of ongoing care from hospital^a^	63 (12.6)	152 (30.4)	285 (57.0)
Details of ongoing care to be provided by GP^a^	133 (26.6)	157 (31.4)	210 (42.0)
Information on when to refer back^a^	231 (46.2)	165 (33.0)	104 (20.8)
Information on possible late effects^a^	291 (58.2)	130 (26.0)	79 (15.8)

**Frequency of providing care related to:**			
Management of treatment-related side effects	61 (12.2)	182 (36.4)	257 (51.4)
Management of psychological symptoms	57 (11.4)	164 (32.8)	279 (55.8)
Lifestyle health care	41 (8.2)	183 (36.6)	276 (55.2)
Advice concerning work and/or finances	146 (29.2)	204 (40.8)	150 (30.0)

a ‘Almost’ always was provided as an option in place of ‘don’t know’ for these questions. The options were rarely/never/sometimes/often/almost always, whereas the options for the other questions were rarely/never/don’t know/sometimes/often.

Most GPs felt that the provision of standardised cancer treatment summaries and survivorship care plans would improve their ability to provide care (*n* = 380, 76%); only a few felt they would not help (*n* = 17, 3%) and some were unsure (*n* = 103, 21%) (data not shown).

Similar numbers of GPs conducted cancer care reviews opportunistically and by offering a specific review appointment with themselves or their practice nurse (Table 3). Forty per cent used a template, mostly developed locally. However, only half of responders felt the cancer care review was useful (53%), with a few feeling it was not useful (10%). GPs who used a template, along with those who made specific appointments for cancer care reviews, were more likely to report that it was useful/very useful than those who did not use a template (OR 1.48 [95% CI =1.02 to 2.12], *P* = 0.035) or completed them opportunistically (OR 3.27 [95% CI = 2.20 to 4.86], *P*<0.0005) (data not shown). The effect of having specific appointments remained significant after adjustment for use/no use of a template (OR 3.20 [95% CI = 2.15 to 4.77], *P*<0.0005) (data not shown).

**Table 3.** Cancer care reviews: current practice and views

**Conduct**	***n* (%)**
Opportunistically, face to face	225 (45)

By offering a specific appointment with a GP	206 (41)

By offering a specific appointment with a practice nurse	13 (3)

By telephone	39 (8)

Never done/don’t know	17 (3)

**Use of a template in a cancer care review^a^**	
**No**	301 (60)
**Yes**	199 (40)
The Macmillan template	8 (2)
A template provided by local clinicians or CCG	82 (16)
A template developed in the practice	91 (18)
Don’t know	18 (4)

**Usefulness**	
Not useful	48 (10)
Not sure	187 (37)
Useful	219 (44)
Very useful	46 (9)

CCG = clinical commissioning group.

a Five hundred GPs answered ‘yes’ or ‘no’; 199 answered ‘yes’ to use of a template .

### Care beyond cancer treatment

**Figure 2.**
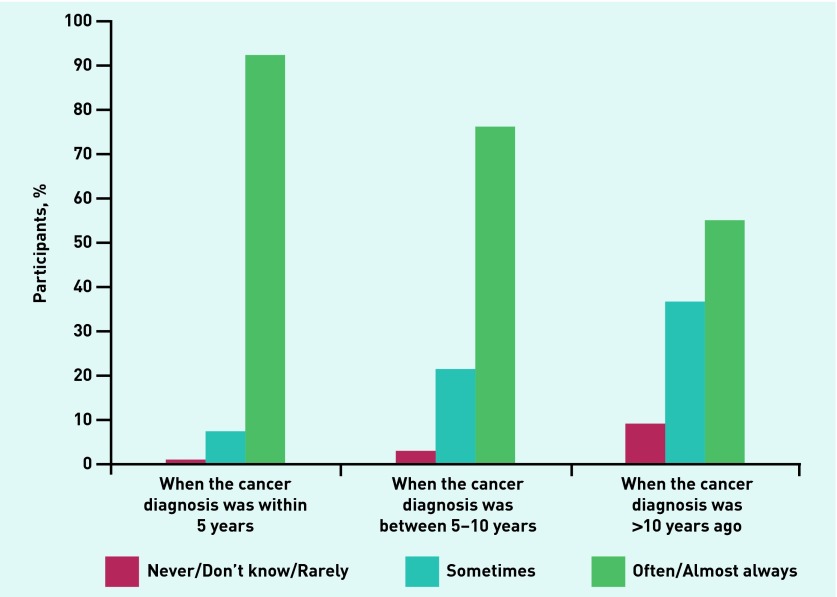
***GPs’ awareness of cancer diagnosis.***

Most GPs reported they would be aware if a consulter had had a cancer diagnosis within 5 years (92%) of the consultation; however, this fell to about half when the cancer diagnosis had taken place >10 years previously (55%) (Figure 2).

Although most GPs reported knowledge of associations between types of cancer treatment and reduced bone health (hormone therapy *n* = 417, 83%; chemotherapy *n* = 338, 68%; radiotherapy *n* = 290, 58%), fewer reported knowledge of similar associations with reduced cardiovascular health (hormone therapy *n* = 267, 53%; chemotherapy *n* = 249, 50%; radiotherapy *n* = 200, 40%). Additionally, fewer than half of the GPs reported often considering a previous history of cancer or cancer treatment when assessing bone health; this was significantly less when assessing cardiovascular health (*n* = 198, 40% versus *n* = 104, 21% respectively, OR 4.76 [95% CI = 3.07 to 7.64], *P*<0.001) (data not shown).

Although only half of the GPs (*n* = 252, 50%) felt often/almost always clear about their role providing care to people living beyond cancer, most believed that secondary and primary care working together should be responsible for managing cardiovascular and bone health (*n* = 385, 77% and *n* = 406, 81% respectively). Some GPs believed it should be primary care alone (*n* = 101, 20% and *n* = 76, 15% respectively), and fewer than 3% opted for secondary care alone (data not shown).

### Training, education, and knowledge

Half the GPs (*n* = 250, 50%) reported having some previous training in general care of people after cancer treatment. Just under one-third had also had some training specifically related to bone or cardiovascular health (*n* = 158, 32% and *n* = 107, 21% respectively). Of all those GPs who reported having any previous training (*n* = 274), most had either undertaken self-directed learning (*n* = 113, 41%) or attended meetings or lectures (*n* = 84, 31%). A small number had completed courses (*n* = 35, 13%) or clinical attachments (*n* = 25, 9%) (data not shown).

Most GPs were keen to undertake further education on cardiovascular (86%) and bone (82%) consequences following treatment, and the management of treatment-related side effects (76%). Fewer felt that they needed further education in the management of psychological symptoms (52%), advice concerning work and/or finances (36%), or lifestyle health care (23%) (Table 4).

**Table 4.** Areas of desired further education

**Area**	***n* (%)**
Management of cardiovascular consequences following treatment	430 (86)
Management of bone consequences following treatment	411 (82)
Management of treatment-related side effects	381 (76)
Management of psychological symptoms	260 (52)
Advice concerning work and/or finances	180 (36)
Lifestyle health care	116 (23)

## DISCUSSION

### Summary

These findings show that the GPs surveyed were generally confident managing treatment-related side effects and psychological symptoms, as well as providing lifestyle advice for people who have recently completed active cancer treatment. They were less confident giving advice concerning work and finances, and provided this information less frequently.

The GPs felt that more information and communication in the form of cancer treatment summaries and survivorship care plans would improve their ability to provide quality cancer care. Although most were providing cancer care reviews, more than half of these are undertaken opportunistically or via telephone consultations and without using templates, and just over one-third of GPs were uncertain about their usefulness.

Most felt that primary and specialist care could jointly manage bone and cardiovascular health consequences. However, many reported being unaware of a previous diagnosis of cancer and did not routinely consider a previous history of cancer when assessing bone or cardiovascular health. Encouragingly, most GPs had an appetite for further education to improve their knowledge and expertise about the management of cancer treatment-related side effects and cardiovascular and bone consequences.

### Strengths and limitations

Using an established research company enabled the views of GPs across each NHS region in England to be sampled, and a large sample of 500 responders was achieved. However, these participants are drawn from a group of GPs interested in taking part in research and, as such, may not be entirely representative of GPs in England. The sample also included more male GPs, GP partners, and trainers, and fewer full-time GPs, than the national average. The findings should, therefore, be generalised to national level with some caution. Furthermore, the survey may be susceptible to response bias; for example, it could be that better-educated GPs or those with a greater interest in the care of survivors of cancer were more likely to respond. In addition, the findings on current practice are self-reported and may differ from actual behaviour.

### Comparison with existing literature

Despite the efforts of the recent NCSI strategies, the findings presented here are not substantially improved from those reported following a similar, but smaller, online survey of GPs in England (*n* = 200) conducted in 2009;^19^ there was no improvement in the proportion conducting a cancer care review opportunistically rather than routinely.

There was also little improvement in the communication from expert care, particularly at the completion of active cancer treatment, and similar findings have been found in North American surveys of primary care physicians.^20^,^24^,^25^ This is despite evidence that interventions improving communication between primary and secondary care have been shown to increase GP involvement in cancer care.^26^

Most responders reported a willingness to accept either shared or sole responsibility for the routine follow-up care of their patients; primary care physicians in North America^27^ and GPs in the Netherlands^28^ have demonstrated similar attitudes. Some studies have only identified this willingness when the GPs were provided with a survivorship care plan, or in consultation with a long-term specialist follow-up programme.^11^,^29^ However, most studies in this area have focused on caring for people as they complete treatment for breast or colorectal cancer. It may be that GP willingness depends on the specific type of cancer, its complexity, and the type of the follow-up tests required; for instance, GPs may have more concerns about caring for survivors of childhood cancer.^11^ A number of studies have also reported disagreement between GPs and oncologists regarding the ideal model of follow-up care, with some oncologists not favouring increased GP involvement^30^ and some GPs preferring main responsibility to remain with oncologists.^31^ These attitudinal barriers could impede implementation of new models of shared care, but may be more prevalent in healthcare systems such as that in the US.^30^

The willingness of GPs to work more closely with secondary care on the follow-up role may also be hampered by a perceived lack of specialist knowledge.^14^ In contrast with the earlier English survey,^19^ this study found an enthusiasm for further training, particularly around the impact of late effects of cancer treatment on bone and cardiovascular health. This may be due to the recent influence of the NCSI in the UK — this contrasts with reports from North America where, although primary care physicians desire to be closely involved in delivering survivorship care, many felt unprepared to both evaluate and manage the long-term effects of their survivors of cancer.^11^,^20^

The resonance of the study findings with results from similar surveys undertaken in North America and Western Europe also suggests that the issues around caring for people living with, and beyond, cancer are universal. Against the backdrop of increasing and ageing populations, along with improved cancer detection and treatment, patients are living with their disease for longer. A recent editorial in *Lancet Oncology* suggested that ‘provision for cancer patients [is] a priority in primary care reforms’,^32^ and highlighted the need for more patient-centred and integrated primary and secondary care. This is supported by the results of this study, in which almost all GPs (97%) agreed that primary care should play a role in the care of patients living beyond cancer, and many felt increased communication between primary and secondary care would improve their ability to provide quality cancer care.

### Implications for practice

Better dissemination of currently available survivorship care resources would encourage their systematic application.^20^ Timely use of cancer treatment summaries would enhance communication between specialist and generalist care, and appropriate development of survivorship care plans would enhance a patient-centred approach. Optimising the utility of current cancer care reviews by encouraging the development of local templates and planned appointments may also enable GPs to feel more confident about their role, and would usefully provide an opportunity for patients to discuss their future care with their GP. This could be achieved by encouraging GP practices to adopt a proactive, rather than reactive, approach to cancer care reviews, through the development of local templates; this appeared to be favoured by GPs as well as by specialists encouraging patients to make an appointment to see their GP at the point of discharge after completion of treatment.^33^

It may also be useful for GPs to make contact with a patient at the time of diagnosis as acknowledgement of the diagnosis and to offer general support, thereby promoting an integrated approach to care.^34^ Integrated care, with seamless transitions between care providers, could also be facilitated by patient-held or electronic resources; there is some recent evidence for the usefulness of web-based survivorship care plans for adult survivors of childhood cancer.^35^,^36^ Although these approaches are all highly desirable, they bring with them resource implications for primary care. In the current context of English primary care struggling to meet current demand, and training places not being filled, the British Medical Association has recently recommended that practices should receive resources to provide extra services.^37^

Although most cancer survivorship work has focused on people living with, and beyond, the most common adult cancers — namely breast, prostate, and colorectal cancer — with reasonable outcomes, survivors of childhood cancer are an important group for the number of life years gained. Most will seek care from primary care, which will need appropriate guidelines and tools to support that. Shared-care models have been piloted in the Netherlands;^35^ similar models may have value in other countries. GPs in remote areas may need more specific guidelines and tools to support shared care.

Late effects of cancer treatment on cardiovascular health are also becoming a priority area as more radiotherapy and combination, adjuvant, and targeted chemotherapies are delivered. Rapid improvements in outcomes from childhood cancer treatment have led to many more adult survivors at increased cardiovascular risk, as well as older adult survivors including those with pre-existing cardiovascular disease. This study showed that, although GPs often did not consider a previous diagnosis of cancer when discussing cardiovascular and bone health, most had an appetite for further education to improve their expertise in the management of cancer treatment-related side effects and cardiovascular and bone consequences.

In conclusion, GPs need efficient tools and appropriate education to provide high-quality care for people living with the consequences of cancer and its treatment. Interventions should focus on improving communication between primary and secondary care, raising awareness of physical and psychological consequences, optimising existing resources, and enhancing knowledge of late effects and how best to manage them.
